# *MMP1 *bimodal expression and differential response to inflammatory mediators is linked to promoter polymorphisms

**DOI:** 10.1186/1471-2164-12-43

**Published:** 2011-01-19

**Authors:** Muna Affara, Benjamin J Dunmore, Deborah A Sanders, Nicola Johnson, Cristin G Print, D Stephen Charnock-Jones

**Affiliations:** 1 Department of Pathology, University of Cambridge. Tennis Court Road, Cambridge, CB2 1QP, UK; 2 Department of Obstetrics and Gynaecology, University of Cambridge. The Rosie Hospital, Robinson Way, Cambridge CB2 0SW, UK; 3 Department of Molecular Medicine and Pathology, School of Medical Sciences, University of Auckland, Private bag 92019, Auckland, New Zealand; 4 National Institute for Health Research, Cambridge Comprehensive Biomedical Centre, Box 277, Hills Road, Cambridge, CB2 0QQ, UK

## Abstract

**Background:**

Identifying the functional importance of the millions of single nucleotide polymorphisms (SNPs) in the human genome is a difficult challenge. Therefore, a reverse strategy, which identifies functionally important SNPs by virtue of the bimodal abundance across the human population of the SNP-related mRNAs will be useful. Those mRNA transcripts that are expressed at two distinct abundances in proportion to SNP allele frequency may warrant further study. Matrix metalloproteinase 1 (MMP1) is important in both normal development and in numerous pathologies. Although much research has been conducted to investigate the expression of *MMP1 *in many different cell types and conditions, the regulation of its expression is still not fully understood.

**Results:**

In this study, we used a novel but straightforward method based on agglomerative hierarchical clustering to identify bimodally expressed transcripts in human umbilical vein endothelial cell (HUVEC) microarray data from 15 individuals. We found that *MMP1 *mRNA abundance was bimodally distributed in un-treated HUVECs and showed a bimodal response to inflammatory mediator treatment. RT-PCR and MMP1 activity assays confirmed the bimodal regulation and DNA sequencing of 69 individuals identified an *MMP1 *gene promoter polymorphism that segregated precisely with the *MMP1 *bimodal expression. Chromatin immunoprecipation (ChIP) experiments indicated that the transcription factors (TFs) ETS1, ETS2 and GATA3, bind to the MMP1 promoter in the region of this polymorphism and may contribute to the bimodal expression.

**Conclusions:**

We describe a simple method to identify putative bimodally expressed RNAs from transcriptome data that is effective yet easy for non-statisticans to understand and use. This method identified bimodal endothelial cell expression of *MMP1*, which appears to be biologically significant with implications for inflammatory disease. (271 Words)

## Background

Numerous strategies have been used in an attempt to sift through the vast amounts of data produced from microarray expression studies [1-4]. There has been much interest given to the identification of bimodally expressed mRNA transcripts, particularly in the context of cancer, where two distinct populations of patients can be defined by differing levels of a set of specific transcripts. These make excellent candidate biomarkers and often tend to show good correlation between transcript and protein abundance [5]. To this end, statistical approaches using mixture-model based clustering combined with either Akaike information criterion (AIC) or the Bayesian informatics criterion (BIC) have frequently been applied [6-9]. One method based on systematic classification of gene expression profiles has been applied to over 2,000 microarray samples [10]. These methods have a strong theoretical base and have proven successful in identifying bimodality. However, they do depend on the investigator having a relatively high level of statistical understanding [7]. In this study we suggest a simple screening approach based on hierarchical clustering to identify bimodally expressed transcripts from microarray expression data that can be used alongside more complex approaches. While this method is not motivated by statistical theory, it appears to work well and is easily understood by laboratory scientists with only basic statistical training, who are in a good position to immediately follow up their results experimentally.

The matrix metalloproteinase, MMP1, is one of the most abundant proteases in the matrix metalloproteinase family. It is capable of degrading type I, II and III collagens, and is one of only four MMPs able to degrade triple helical collagens. It therefore plays a pivotal role in extracellular matrix (ECM) remodelling in both normal development and pathology [11]. MMP1 is tightly regulated at both the transcriptional and post-translational levels. It is produced as a zymogen that is activated by serine proteases and its activity is regulated by inhibitors such as the tissue inhibitors of metalloproteinase's (TIMPs), which compete with the substrate for the enzyme active site [12].

MMP1 plays a clinically important role in inflammatory disease, and has been implicated in numerous pathological processes including wound healing [13], tumour metastasis [14] and arthritis [15]. The *MMP-1 *gene [11] contains a 1G/2G polymorphism in its promoter at -1607 from the transcriptional start site [16]. This polymorphism has been associated with increased *MMP-1 *expression in several cell types including; melanoma, stromal fibroblast, MCF-7/ADR breast cancer cells [16-19], and with several pathologies including: tumour metastasis [20,21], arthritis [22,23], periodontitis [24], chronic obstructive pulmonary disease [25] and cardiovascular disease [26,27].

Using our simple clustering method to examine RNA transcript abundance in HUVECs isolated from 15 different human individuals, we identified *MMP1 *as one of a small group of RNAs expressed in a bimodal manner in both un-treated endothelial cells, and in endothelial cells treated by inflammatory mediators. Our results suggest that the regulation of *MMP1 *expression is a complex process that is modulated by a promoter polymorphism around the binding sites for several TFs including ETS1, ETS2 and GATA3.

## Results

### Identification of mRNA transcripts with bimodal expression patterns among a set of individuals

**Figure 1:**
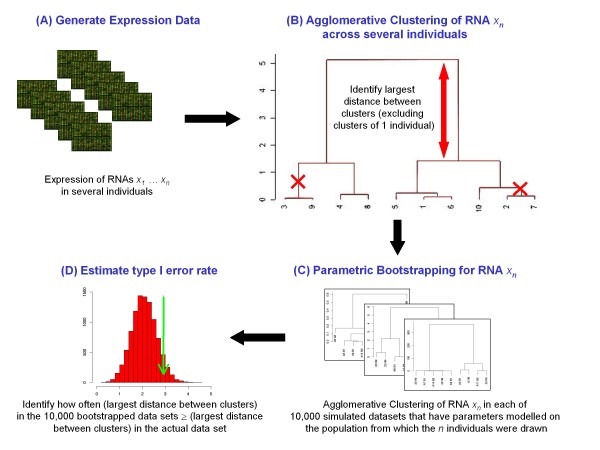
**Flow diagram of method to identify bimodally expressed transcripts from expression data**. (A) Transcript abundance is quantified by microarray or RNAseq techniques. (B) On a transcript-by-transcript basis, agglomerative clustering across the dataset is carried out. The algorithm starts by assigning the same number of clusters as individuals (in this example 10 clusters were assigned since there are 10 individuals). The clusters are then progressively merged by combining the two most similar clusters, using Wards method to calculate the distance between clusters and Euclidian distance to calculate dissimilarities between the individuals. The distances between the merging clusters are recorded by the algorithm as branch "heights". The height values at either side of the dendrogram are removed to exclude transcripts that falsely appear to be bimodally expressed due to a single outlying individual. The maximum remaining branch height value (indicated by the red arrow) is identified for each transcript, which represents the greatest distance between the any two clusters of individuals, and is used a surrogate marker for the degree of bimodal expression for that particular transcript. (C) To estimate the probability of transcripts appearing to be bimodally expressed due to chance alone, for each transcript we make a maximum likelihood estimate of the parameters of the distribution of this transcript's abundance across the population from which the individuals being studied have been drawn. We use these parameters to generate 10,000 simulated datasets, each of which is clustered as described in (B) above. (D) In the 10,000 clusters formed from the bootstrapped data sets for this transcript, we identify how commonly the largest distance between clusters ≥ the largest distance between clusters in the actual data set. This information is shown graphically and is used generate an empirical p-value as an estimate of type I error rate.

Bimodally or multimodally expressed mRNA transcripts were defined as those transcripts for which two or more distinct populations of expression values were observed among a set of individuals. To identify and visualise bimodally expressed transcripts, we devised a simple algorithm (written as a script in the statistical language 'R'; Additional File 1) based on unsupervised agglomerative hierarchical clustering. The algorithm can be used as either a simple 'R' script, or for use in graphical user interface it can be supplied as a GenePattern module on request (http://www.broadinstitute.org/cancer/software/genepattern/). It is illustrated schematically in Figure 1 and described in the methods section. Briefly, on a transcript-by-transcript basis, agglomerative hierarchical clustering across the dataset was carried out. The maximum cluster branch height identified for each transcript was approximately proportional to the greatest distance between the any two clusters of individuals, and is used here a surrogate marker for the degree of bimodal expression. To estimate the probability of transcripts appearing to be bimodally expressed due to chance alone we used a parametric bootstrapping method. Related methods where trees are constructed from re-sampled data have been used previously to assess the reliability of clusters in gene expression data [28]. As is often the case with microarray transcript abundance data, our log-transformed data approximated a normal distribution. Therefore, for each transcript we made a maximum likelihood estimate of the parameters of a normally distributed population from which the sample of individuals being studied may have been drawn. These parameters (mean and standard deviation) were then used to generate 10,000 simulated datasets for the transcript, each of which was clustered as described above. From the 10,000 clustering results we identified how frequently the largest distance between clusters ≥ the largest distance between clusters in the actual data set. This information is used generate an empirical p-value as an estimate of type I error rate.

**Figure 2:**
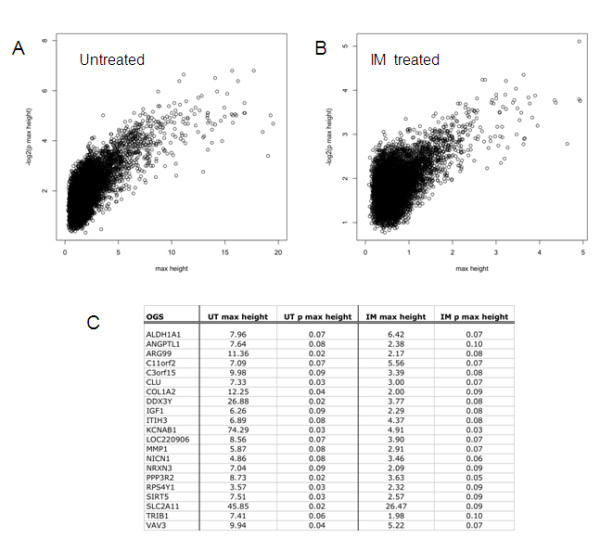
**Microarray data of approximately 16,000 transcripts in un-treated HUVECs from 15 individuals and IM-treated HUVECs from 9 individuals were analysed separately using the clustering method described in this paper**. The maximum branch height (max height, an estimate of the degree of bimodal expression) for each transcript was plotted against the -log2 empirical p-value (an estimate of the frequency of that transcript appearing to be bimodally expressed due to chance alone) for the un-treated data set (A) and the IM-treated data set (B). The 21 RNA transcripts for which the empirical p-values were ≤ 0.1 in *both *the un-treated and IM-treated data sets were identified and are listed alphabetically in panel (C).

This algorithm was applied to two RNA microarray data sets: (i) data from HUVECs from 15 different human individuals cultured to passage 4 in standard conditions (the untreated data set; UT) and (ii) data from passage 4 HUVECs from nine different human individuals cultured with a cocktail of 10 ng/ml TNF-α, IL-1β and IL-8 for 24 hours (the IM-treated data set; IM). The bimodally expressed RNAs found in the UT and IM HUVECs are listed in Additional File 2. The relationship between the maximum branch height (an estimate of the degree of bimodal expression) and the -log2 transformed empirical p-value (an estimate of the frequency of a transcript appearing to be bimodally expressed due to chance alone) is shown in Figure 2A-B. In each of the un-treated and IM-treated data sets, a relatively small group of transcripts with high maximum branch height and high -log2 p were identified. We decided to accept an estimated type I error rate of 10% for each of these data sets, and found there were 21 RNA transcripts for which the empirical p-values were ≤ 0.1 in *both *the un-treated and IM-treated data sets (Figure 2C). A table of features for each of the 21 shortlisted RNA transcripts is given in Additional file 3.

These shortlisted transcripts were further assessed using several strategies including; i) visual inspection of histograms on a gene by gene basis (an R script used to generate these is given in Additional File 4); ii) evaluation of the associated bootstrap p-values obtained during clustering and iii) consideration of additional information of biomedical interest. We were especially interested in the presence of promoter SNPs, which may in theory cause bimodal RNA expression patterns, for example using the *SNPer *[29] or *rSNPs *[30] databases. Based on all these considerations, subset of transcripts were selected to take forward for further investigation; *DDX3Y *(a Y-chromosome encoded RNA, which should segregate with gender), *MMP1 *and *SLC2A11 *(biologically interesting TNFα and IFNγ targets, which are important in inflammation).

### *DDX3Y *is differentially expressed on the basis of its location on the Y-chromosome

**Figure 3:**
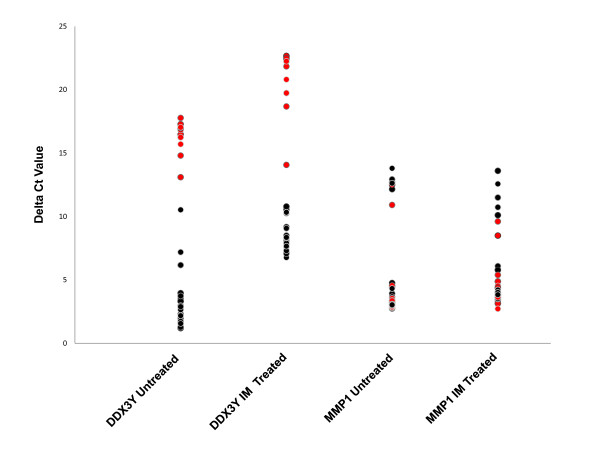
**Quantitative PCR of transcripts DDX3Y and MMP1 in 29 donor isolates under untreated (UT) and inflammatory mediator treated (IM) conditions**. Donors of female gender are shown in red and donors of male gender are shown in black. Only DDX3Y appears to segregate with gender. Delta Ct values are calculated as Ct of target gene of interest (DDX3Y or MMP1) relative to the Ct value of the internal control (18S ribosomal RNA). Low delta Ct values represent high basal levels of expression and vice versa.

The pool of individuals examined was expanded and the abundance of *DDX3Y*, *MMP1 *and *SLC2A11 *mRNA in HUVECs from 29 additional individuals cultured in both UT and IM conditions was analysed using quantitative Reverse Transcription-PCR (qRT-PCR). We confirmed in this new group of individuals a bimodal expression pattern for *DDX3Y *using q RT-PCR. *DDX3Y *is encoded by a Y-chromosome gene and, as expected, its expression segregated with the gender of the individual from which the HUVEC were isolated in both the IM and UT data (Figure 3).

### *MMP1 *is differentially expressed and differentially active in endothelial cells

**Figure 4:**
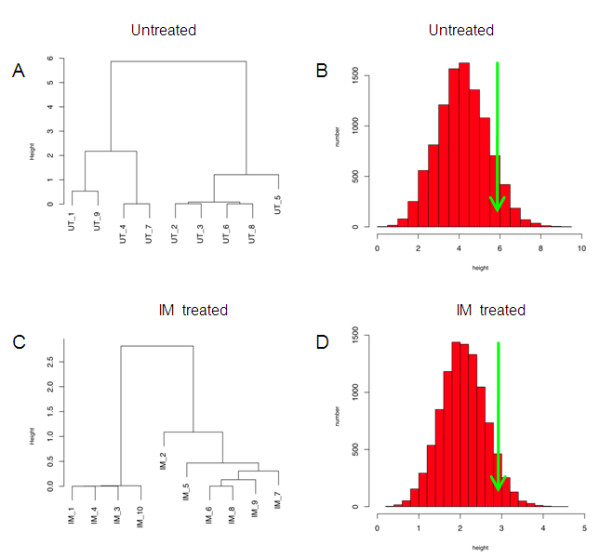
**Dendrograms and histograms for MMP1 in untreated and IM treated HUVECS**. Dendrograms of *MMP1 *RNA expressions are shown for the un-treated (A) and IM-treated (C) HUVECs. Histograms showing the frequency (y-axis) of maximum branch height (x-axis) across 10,000 simulated *MMP1 *transcript datasets, each with parameters similar to the estimated parameters of the population from which the actual *MMP1 *data set was drawn, are shown (B and D). Green arrows indicate the maximum branch height from cluster analysis of the actual data sets. In both un-treated and IM-treated HUVECs, the maximum clustering branch height for *MMP1 *exceeded the maximum clustering branch height identified in 90% of the simulated data sets.

**Figure 5:**
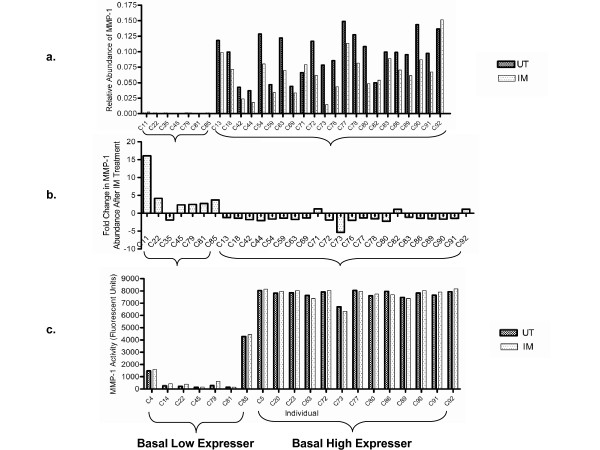
**Differential expression of *MMP1 *in un-treated and inflammatory mediator-treated HUVECs isolated from 29 separate individuals**. (a) Quantitative RT-PCR measuring *MMP1 *abundance in un-treated (UT) and inflammatory mediator-treated (IM) conditions identified two distinct populations; HUVECs isolated from seven individuals had low *MMP1 *mRNA abundance regardless of UT or IM culture conditions (labelled as Basal Low Expressers), while HUVECs isolated from the other 22 individuals had relatively higher *MMP1 *mRNA abundance regardless of UT or IM culture conditions (labelled as Basal High Expressers) (P < 0.0001, Mann Whitney non parametric test). (b) To illustrate the regulation of *MMP1 *mRNA abundance by inflammatory mediator-treatment, the fold change in *MMP1 *abundance in IM -vs- UT conditions is shown. In six out of the seven Basal Low Expressers *MMP1 *abundance was increased in response to IM treatment. In contrast, in 19 out of the 22 Basal High Expressers *MMP1 *abundance either did not change significantly or fell slightly in response to IM treatment. Both the difference in mRNA abundance between the untreated and the inflammatory mediator treated conditions and the differential response between the high and low basal expressers to inflammatory mediator treatment showed statistical significance (Paired t-test, P < 0.0001 and Mann Whitney nonparametric test, P = 0.002 respectively). (c) Total MMP1 enzymatic activity was measured in a subset of the HUVEC isolates. In general low basal expressers have lower enzyme activity than high basal expressers.

*MMP1 *was of particular interest since it encodes a biologically and clinically important enzyme, and since analysis of the *rSNPs *database identified several common SNPs in the *MMP1 *gene promoter within 2,000 bp upstream of the start of transcription (data not shown). Dendrograms for MMP1 in un-treated and IM-treated HUVECs, along with histograms of the maximum cluster branch height in each of 10,000 parametric bootstrap data sets (to estimate the probability of transcripts appearing to be bimodally expressed due to chance alone) are shown in Figure 4. Quantitative RT-PCR from 29 additional individuals confirmed in this new group of individuals a bimodal expression pattern for *MMP1*. We identified two distinct populations; (i) HUVECs isolated from 7 of the 29 individuals had low *MMP1 *mRNA abundance (Figure 5a), however in 6 of these 7 individuals, *MMP1 *mRNA abundance was significantly increased by culture in IM conditions (Figure 5b). (ii) The remaining 22 individuals had relatively higher *MMP1 *mRNA abundance regardless of UT or IM culture conditions (Figure 5a). In the high *MMP1 *expressing HUVECs isolated from these 22 individuals, the abundance of *MMP1 *mRNA was either not significantly affected or was decreased by IM culture conditions (Figure 5b). This differential response to inflammatory mediator treatment was striking and we sought to understand the underlying mechanisms.

To investigate whether the bimodal expression of *MMP1 mRNA *was also evident at the level of MMP1 enzymatic activity, this activity was measured in 20 HUVEC isolates. The activity of MMP1 was low in those HUVEC cultures that had low *MMP1 *RNA abundance and high in those HUVEC cultures that had high *MMP1 *RNA abundance (Figure 5c).

### *MMP-1 *gene promoter polymorphisms segregate with MMP1 expression and enzymatic activity

**Figure 6:**
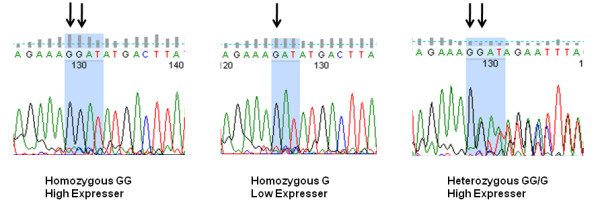
**Sequencing of the -1607 *MMP-1 *promoter polymorphism, illustrating the homozygous and heterozygous genotypes**. Insertion of an additional G creates the consensus sequence for the ETS binding site, GGA (indicated by blue shading).

A 1G/2G deletion/insertion polymorphism at position -1607 in the *MMP-1 *promoter has been associated with differential expression of this gene in fibroblast and melanoma cells, with the 2G genotype associated with higher basal levels of *MMP1 *mRNA [16]. This polymorphism occurs within a consensus binding site for the ETS family of transcription factors [16,18]. Therefore, direct DNA sequencing of the *MMP1 *promoter was carried out to determine whether the *MMP1 *mRNA abundance and activity profiles segregated with this promoter polymorphism. We determined *MMP1 *mRNA level, MMP1 enzymatic activity and *MMP1 *promoter genotypes in HUVECs from 69 different individuals. Figure 6 and Table 1 illustrate the three genotypes observed in these individuals. Of the 69 individuals, 76% were heterozygous at the site of the promoter polymorphism, 15% were homozygous for the 1*G *allele, while only 9% were homozygous for the 2*G *allele. All 1*G *homozygous individuals segregated with low basal expression and activity of MMP1, whereas all but one of the heterozygotes and homozygous 2*G *individuals segregated with high basal expression and activity of MMP1.

**Table 1:** Segregation of genotype for the -1607 *MMP-1 *promoter polymorphism with basal abundance of *MMP-1 *transcript

	High Basal Expression	Low Basal Expression	Total
**Homozygous 1G SNP**	0	11	**11**

**Homozygous 2G SNP**	5	1	**6**

**Heterozygous 1G/2G SNP**	52	0	**52**

**Total**	**57**	**12**	**69**

### Elevated *MMP1 *expression induced by inflammatory mediator in 1*G *homozygous cells is not due to greater overall activity of pro-inflammatory signalling pathways

**Figure 7:**
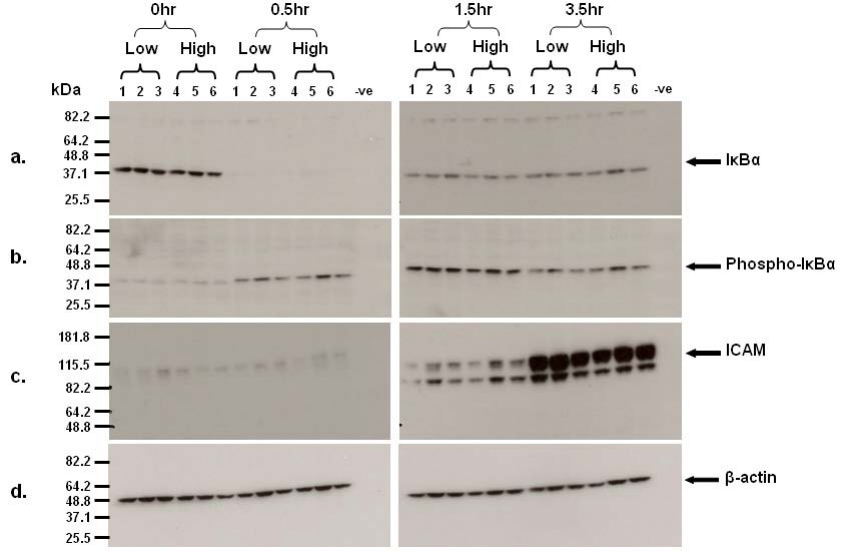
**Immunoblot illustrating inflammatory signalling responses in 3 isolates with low and high basal expression of *MMP-1 *(labelled isolates 1, 2, 3 and 4, 5, 6 respectively)**. Low = low basal expressers and High = high basal expressers. Time in hours for each group of low and high basal expressers is given at the top of the figure. (a) IκBα signalling over 3.5 hours after the addition of an inflammatory mediator cocktail containing 10 ng/ml each of TNF-α, Il-1β and Il-8. (b) Phosphorylated IκBα signalling over the time course. (c) ICAM signalling over the time course. Both the high expressers and low expressers show similar levels of IκBα, phospho- IκBα and ICAM over the time course.

It was possible that the differential responses to IM treatment we observed between individuals with low *MMP1 *basal expression (homozygous 1*G *individuals) and high *MMP1 *basal expression (heterozygotes and homozygous 2*G *individuals) were simply due to differential activity of the signalling pathways that mediate inflammation. Therefore, molecules known to be downstream of inflammatory mediator signalling were assessed as biomarkers of inflammatory pathway activity in individuals with low and high *MMP1 *basal expression. Protein expression levels in ICAM1, IкBα and phospho-IкBα were measured in HUVEC lysates from three individuals with low basal *MMP1 *mRNA and three with high basal *MMP1 *mRNA, after treatment with 10 ng/ml IL-1β, TNF-α and IL-8 for up to 3.5hrs. Figure 7 shows abundance of ICAM-1, total and phospho-IкBα over the 3.5hr period. Two-way analysis of variance (ANOVA) revealed that there was no significant difference in ICAM1 or total and phospho-IкBα signal between the high and low expressers at all time points (P = 0.8, 0.7 and 0.2 respectively). These results suggest that there is not a large systematic difference between the inflammatory signalling pathways related to this polymorphism.

### Ets1, Ets2, Fos and GATA3 are potential mediators of the different expression levels of *MMP1 *mRNA in 1G -vs- 2G individuals

**Figure 8:**
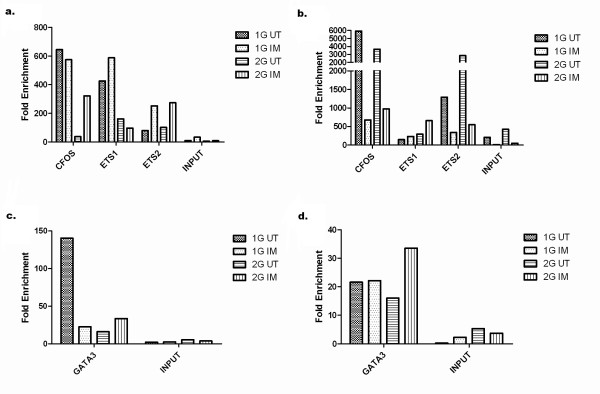
**Fold enrichment of transcription factor binding to the region of the -1607 MMP1 promoter polymorphism**. (a) Fold enrichment of c-Fos, ETS1 and ETS2 binding at the region of the -1607 promoter polymorphism relative to a region 5600 bases upstream of the promoter polymorphism. (b) Complete replication of experiment in 5a using cells from different 1G and 2G individuals. (c) Fold enrichment of GATA3 binding at the region of the -1607 promoter polymorphism relative to a region 5600 bases upstream of the promoter polymorphism. (d) Replication of experiment 5c. Input control refers to enrichment in the absence of any precipitating antibody.

To determine whether differential binding of transcription factors to the *MMP1 *promoter region containing the -1607 polymorphism is a potential mechanism for the differential response of *MMP1 *to IM treatment, the TFSEARCH algorithm [31] was used to identify putative transcription factor binding sites in this region. This analysis revealed in addition to the ETS binding domain spanning the polymorphic region, AP-1 and GATA3 binding sites were located 44 bp and 5 bp respectively downstream from the polymorphism. To determine whether these TFs actually bind to this region of the *MMP1 *gene promoter, immunoprecipitation using antibodies against c-fos, ETS1, ETS2 and GATA3 was carried out in HUVECs isolated from two individuals of 1G genotype and in HUVECs isolated from two individuals of 2G genotype cultured in both IM and UT conditions. Quantitative PCR was used to measure the enrichment of the immunoprecipitated region (157 bp region, adjacent to an ETS, GATA3, AP-1 and NFKB binding site), relative to a 173 bp region positioned 5600 bases upstream of the polymorphism that contained no relevant motifs. DNA precipitated by anti-c-fos, anti-ETS1, anti-ETS2 and anti-GATA3 antibodies was enriched for the *MMP1 *promoter region containing the polymorphism, relative to the control upstream region and relative to the un-immunoprecipitated material in all four individuals and in cells cultured in UT and IM conditions (Figure 8). The aim of this experiment was to identify potential TF binding around the polymorphism, not to compare the degree of enrichment between IG and 2G individuals or between UT or IM cultured cells, which was impossible due to the small study size and variable degree of enrichment between individuals (Figure 8). Nevertheless, it was interesting to observe that, consistent with the previously published role of ETS1 in *MMP-1 *induction [16], ETS1 binding was reproducibly enriched in the 1G isolates treated with IM.

## Discussion

The significance of non-coding polymorphisms in pathology is being increasingly recognised, with much research being carried out to identify the functional importance of the millions of SNPs mapped to date in the human genome [32]. This study suggests a complementary strategy, whereby we first identified those transcripts that showed bimodal expression levels, and then identified the polymorphism responsible for this differential expression. Clustering methods [6] and statistical methods [7,10,33] have previously been used to identify bimodal expression in large datasets. These methods have the advantage of being motivated by strong theoretical statistical considerations. However, they also require a moderate level of statistical understanding, and in addition some of these methods can only be easily applied to large-scale meta-analysis of several data sets [10] and may be less suitable for small expression data sets generated in a single laboratory. The approach we suggest is able to be used alongside more complex approaches by laboratory scientists with only basic statistical training, who are in a good position to immediately follow up their results experimentally. We believe that for bioinformatic tasks such as this, providing several complimentary methods that span the continuum of statistical complexity is important in order to bridge the gap between experimental biologists and statisticians.

Our clustering strategy successfully identified the abundance of several mRNA transcripts including *MMP1 *as bimodally distributed in human endothelial cells in both resting and inflammatory mediator-treated conditions. Up-regulated *MMP1 *expression has been associated with many pathologies in which endothelial cells are involved [20,21,26,27]. We sequenced the region spanning the well characterised polymorphism at -1607 from the transcription start site in the *MMP-1 *promoter [16-19] and found that this polymorphism was strongly associated with the bimodal expression observed in the HUVEC dataset. Individuals either homozygous or heterozygous for the 2G polymorphism at -1607 possessed constitutively higher levels of *MMP1 *(over 100 fold relative to the 1G isolates, P < 0.0001), implying a dominant effect of the 2G allele. This contrasts with previous findings in fibroblasts, where marginal differences in the basal levels of *MMP1 *between the 1G and 2G isolates were observed [17,34].

Inflammatory mediator treatment revealed a differential response in MMP1 stimulation between HUVEC isolates homozygous for the 1G allele and those possessing the 2G allele. Whereas *MMP1 *mRNA levels were increased in all isolates homozygous for the 1G allele; in isolates possessing the 2G allele, *MMP1 *mRNA levels were essentially unchanged (Figure 5). Regulation at the level of MMP1 enzyme activity mirrored this response to inflammatory mediator treatment. One possibility is that *MMP1 *expression is at maximum levels in the 2G isolates, even under the basal condition. In leukocytes continuously treated with high doses of inflammatory mediators (50 ng/ml, of TNF-α and Il-1β, 2 or 3 times over 24hrs), *MMP1 *mRNA levels are genotype independent [24]. Whether this is the case in HUVECs remains to be determined.

Chromatin immunoprecipitation (ChIP) was conducted to investigate the potential involvement of TFs that have putative response elements spanning the polymorphism. We found that three TFs bound to this polymorphic region of the *MMP1 *promoter in endothelial cells. Using nuclear extracts from fibroblast and A2058 melanoma cells, Rutter et al. previously demonstrated that recombinant ETS1 binds strongly to the 2*G *promoter and weakly to the 1*G *promoter and that this binding is dependent on cooperation with an adjacent AP1 site at -1602 [16]. Their study along with others have revealed that several members of the AP1 family, including c-JUN, Fra and Fos, are involved in the heterodimmer complexes bound at this cooperative AP1 site [16,35,36]. While the limited ChIP analysis we have performed clearly indicated that GATA3, Fos, Ets1 and Ets2 do indeed bind to this region of the *MMP1 *promoter, further studies using larger numbers of individuals will be required to identify differential binding between genotypes or cell culture conditions.

## Conclusion

Identifying the functional importance of the millions of human SNPs is becoming a major challenge. Simultaneously, the amount of available RNA transcriptome data is rapidly growing, driving scientists to devise new methods to extract the most biologically and clinically useful information from RNA abundance profiles. Therefore, a strategy that identifies functionally important SNPs by virtue of the bimodal abundance across the human population of the associated mRNAs is potentially very useful. Here, we discuss a simple method based on hierarchical clustering to identify bimodally expressed transcripts, which may be used with either microarray or RNAseq data. This method complements more statistically complex approaches and is suitable for use by laboratory scientists with only basic statistical training, who are in a good position to immediately follow up their results experimentally. This strategy identified bimodal endothelial cell expression of several transcripts including *MMP1*, which appears to be biologically significant with implications for inflammatory disease and for understanding the complex relationships between TFs and polymorphic promoter elements.

## Methods

### Cell culture and IM treatment

Umbilical cords were collected after written informed consent and the study was approved by the Cambridge Research Ethics Committee. The population sampled for this study were of unknown demography, with no information obtained during donor collection relating to parental age, ethnicity or familial history of disease. HUVECs were isolated by collagenase digestion, as previously described [37]. Cells were cultured in fully supplemented media without antibiotics (basal EBM-2 with a propriety mix of heparin, hydrocortisone, vascular endothelial growth factor, epidermal growth factor, fibroblast growth factor, 2% foetal calf serum (FCS, Lonza, Cambridge, UK), at 37°C/5% CO_2 _until passage 4. To carry out inflammatory mediator treatment for microarray gene expression profiling, passage 4 HUVECs were treated with a cocktail of 10 ng/ml TNF-α, IL-1β and IL-8 for 24 hours prior to RNA extraction.

### RNA processing and microarray preparation and data processing

RNA was extracted using TRIzol^® ^reagent (Invitrogen, London UK). RNA quality was assessed using the Agilent 2100 bioanalyser. Biotin labelled cRNA was generated and hybridised on the CodeLink Human Uniset 20K microarrays following the manufacturer's instructions (Applied Microarrays, formally supplied by GE Healthcare). CodeLink microarray data was pre-processed to assess array quality using the CodeLink Expression analysis software v4.0. To enable comparable analysis between arrays, normalisation was carried out using the cyclic Loess method [38,39]. The microarray data has been deposited in NCBI's Gene Expression Omnibus (GEO) [40] and can be accessed through GEO series accession number GSE23070.

### Bimodal analysis of microarray expression data

To identify bimodally expressed RNA transcripts, RNA was prepared from passage 4 HUVECs isolated from 15 different individuals and analysed using CodeLink Human Uniset 20K microarrays (the untreated (UT) data set). In addition, passage 4 HUVECs isolated from 9 different individuals were each treated with 10 ng/ml of each of TNF-α, IL-1β, Il-8, and analysed using microarrays as described above (the inflammatory mediator treated (IM) data set). Unsupervised agglomerative clustering was then applied separately to the UT and IM data sets to enrich for multimodality, using R bioinformatic software (freely available at http://cran.r-project.org/). For each transcript, our algorithm recorded the "height" (Euclidian distance) between the clusters. The height values at either end of the cluster dendrogram were discarded to remove cases where the clustering identified a single outlying individual, and the largest remaining height value was used as an indicator of bimodality/multimodality. For those RNAs with signal intensities that were similar across the set of individuals, the height between clusters is likely to be small. However, where there were two or more distinct clusters of expression values among the set of individuals, the height between clusters is likely to be large. In addition, parametric bootstrapping was carried out during the clustering process to identify the likelihood of identifying the given height value for each gene based on chance alone, as summarised in Figure 1 and in the comments within the R script in Additional File 1. To be strictly statistically correct, the permutation p-values should be adjusted for multiple testing. For example the Benjamini & Hochberg procedure could be used to control the false discovery rate by applying the mt.rawp2adjp function of the 'multtest' R package to the p-values produced from the bootstrap procedure described here. However, this is not included in the current iteration of our method, since it does not alter the ranking of the permutation p-values assigned to each RNA, and it appears to be overly stringent since it masks both of the bimodally-expressed RNAs that were experimentally confirmed in our study. Nevertheless, if larger data sets are analysed, from which the degree of bimodal expression and population distribution parameters for each RNA can be estimated more precisely, it may be worth experimenting with various multiple testing control procedures.

### Inflammatory mediator time course and immunoblotting

For the inflammatory mediator time course, passage 4 HUVECs were treated with a cocktail of 10 ng/ml of each of TNF-α, IL-1β, IL-8 for up to 3.5 hours. Whole cell lysates were harvested by scraping in 1X RIPA lysis buffer (Millipore, Watford, UK) with protease inhibitors (Roche, Welwyn Garden City, UK), at time points 0, 0.5, 1.5 and 3.5 hours. Proteins were separated on 12% Tris-glycine SDS-page gels (Invitrogen) and transferred to 0.2 μm nitrocellulose membranes (Invitrogen). All membranes were blocked with 5% skimmed milk in Tris-buffered saline/0.01% Tween^®^20 at room temperature. Blots were probed with antibodies against ETS1 (sc-350) and ETS2 (sc-351) (both from Santa Cruz Biotechnology) and β-actin (Ambion).

### MMP1 activity assay

Total active MMP1 protein abundance was measured using the Fluorokine Human Active MMP1 Fluorescent Assay (R&D Systems). Supernatants were collected from the cell culture of 20 different passage 4 HUVEC isolates, treated with and without an inflammatory mediator cocktail of 10 ng/ml TNF-α, IL-1β, IL-8 for 24hrs. P-Aminophenylmercuric Acetate (APMA) was added to all samples to activate any inactive MMP1. Measurement of MMP1 activity was carried out according to the manufacturer's instructions.

### Sequencing of the MMP1 promoter polymorphism

To characterise the -1607 MMP1 promoter polymorphism, DNA was extracted from HUVEC cell pellets using the DNeasy blood and tissue kit (Qiagen, West Sussex, UK), following the manufacturer's instructions. Genomic DNA (50 ng) was amplified with the following primers: 5'-AACCTATTAACTCACCCTTGT-3' 5'-CCTCCATTCAAAAGATCTTATTATTTAGCATCTCCT-3' [34]. The cycling conditions were as follows: pre-incubation at 94°C for 5 minutes, followed by 35 cycles at 94°C for 30 seconds, 56°C for 30 seconds and 72°C for 1 minute, followed by a final extension at 72°C for 10 minutes. PCR products were diluted 1 in 10 in nuclease free water and directly sequenced using the forward primer at GeneService (Cambridge Science Park, Milton, UK). Amplification of the MMP1 promoter region spanning the -930 and -519 polymorphisms was achieved using the same conditions described above using the following primers: 5'-TTCCAGCCTTTTCATCATCC-3' and 5'-CGGCACCTGTACTGACTGAA-3'. Again the forward primer was used for sequencing.

### Quantitative PCR

cDNA was made from 1 μg of total RNA using the Quantitect reverse transcription kit (Qiagen), following the manufacturers protocol. Quantitative PCR was carried out using the the ABI 7700 sequence analyser (Applied Biosystems, Calafornia, USA). Reactions were carried out using the Applied Biosystems universal master mix according to the manufacturers instructions. The Taqman probe primers used were: *MMP1 *(Hs00233958_m1), *DDX3Y *(Hs00190539_m1), *ETS1 *(Hs00901425_m1), *ETS2 *(Hs00232009_m1), *GATA3 *(Hs00231122_m1), *SLC2A11 *(Hs00368843_m1), *DERP6 *(Hs00209768_m1) and internal control 18S (Hs99999901_s1), all from Applied Biosystems.

### Chromatin immunoprecipitation

Passage 4 HUVECs were either treated with vehicle or an inflammatory cocktail of 10 ng/ml TNF-α, IL-1β, IL-8 for 24 hours. Chromatin was cross-linked by the addition of formaldehyde to a final concentration of 1% for 10 minutes at 37°C. Cells were washed in ice cold phosphate-buffered saline containing 125 mM glycine, 1 mg/ml Pefabloc, 1 μg/ml aprotinin and 1 μg/ml pepstatin A. Chromatin was sonicated and immunoprecipitated using specific antibodies, as described in the ChIP protocol from Upstate Inc. (Charlottesville, VA). The following antibodies were used: ETS1 (sc-350), ETS2 (sc-351), c-Fos (sc-52) and GATA3 (sc-268). All antibodies were from Santa Cruz Biotechnologies. To quantify enrichment of binding, quantitative PCR was carried out on the immunoprecipitated DNA using SYBR Green on the iCycler (Roche). 25 μl reactions with 1 X SensiMixPlus SYBR and fluorescein (Quantace) were carried out according to the manufacturer's instructions. Primers around the *MMP1 *polymorphism were 5'-TCTTTGTCTGTGCTGGAGTA-3' and 5'-CAATTTCCTCATCTAAGTGGCATA-3'. The primers for the region 5600 bases upstream of the promoter were 5'-TGCTTATGTTAGCTGACCAGAC-3' and 5'-AGTATGCGTTGCCTTGTCCT-3'.

## Authors' contributions

Conceived and designed the experiments: CP, DSCJ, MA. Performed the experiments: MA, DS. Analysed the data: MA, CP DSCJ. Contributed to reagents/materials/analysis tools: BD, NJ, CP. Wrote the paper: MA, DSCJ, and CP. All authors have read and approved the final manuscript.

## Supplementary Material

Additional file 1 **R-script**: Source code to identify and visualise bimodally expressed transcripts from a microarray expression dataset. Click here for file

Additional file 2 **Clustering results**: Clustering results for both the un-treated (UT) and IM-treated (IM) HUVEC data set are shown for 10531 RNA transcripts. "OGS" is the official human gene symbol. "max_height" denotes the greatest distance (maximum cluster branch height) between the any two clusters of individuals, and is used a surrogate marker for the degree of bimodal expression. "p_max_height" denotes, for this transcript, the frequency with which this maximum cluster branch height is exceeded in clustering of 1,000 simulated data sets generated by aprametric bootstrapping (an estimate of the probability of each transcripts appearing to be bimodally expressed due to chance alone). Click here for file

Additional file 3 **Table of 21 short listed RNA transcripts**: Table of features for each of the 21 RNA transcripts to help determine selection Click here for file

Additional file 4 **R-script**: To plot histograms. Click here for file
